# Malabaricone-A Induces A Redox Imbalance That Mediates Apoptosis in U937 Cell Line

**DOI:** 10.1371/journal.pone.0036938

**Published:** 2012-05-10

**Authors:** Alak Manna, Piu Saha, Avijit Sarkar, Debanjan Mukhopadhyay, Ajay K. Bauri, Deepak Kumar, Padma Das, Subrata Chattopadhyay, Mitali Chatterjee

**Affiliations:** 1 Department of Pharmacology, Institute of Post Graduate Medical Education and Research, Kolkata, West Bengal, India; 2 Bio-Organic Division, Bhabha Atomic Research Centre, Mumbai, Maharashtra, India; 3 Department of Cell Biology and Physiology, Indian Institute of Chemical Biology, Kolkata, West Bengal, India; University of Illinois at Chicago, United States of America

## Abstract

**Background:**

The ‘two-faced’ character of reactive oxygen species (ROS) plays an important role in cancer biology by acting both as secondary messengers in intracellular signaling cascades and sustaining the oncogenic phenotype of cancer cells, while on the other hand, it triggers an oxidative assault that causes a redox imbalance translating into an apoptotic cell death.

**Principal Findings:**

Using a tetrazolium [{3-(4,5-dimethylthiazol-2-yl)-5-(3-carboxymethoxyphenyl)-2-(4-sulfophenyl}-2H-tetrazolium] based cell viability assay, we evaluated the cytotoxicity of a plant derived diarylnonanoid, malabaricone-A on leukemic cell lines U937 and MOLT-3. This cytotoxicity hinged on its ability to cause a redox imbalance via its ability to increase ROS, measured by flow cytometry using 5-(and-6)-chloromethyl-2′,7′-dichlorodihydrofluorescein diacetate and by decreasing glutathione peroxidase activity. This redox imbalance mediated apoptosis was evident by an increase in cytosolic [Ca^2+^], externalization of phosphatidyl serine as also depolarization of the mitochondrial membrane potential as measured by flow cytometry. There was concomitant peroxidation of cardiolipin, release of free cytochrome *c* to cytosol along with activation of caspases 9, 8 and 3. This led to cleavage of the DNA repair enzyme, poly (ADP-ribose) polymerase that caused DNA damage as proved by labeling with 4′,6-diamidino-2-phenylindole (DAPI); furthermore, terminal deoxy ribonucleotide transferase catalysed incorporation of deoxy uridine triphosphate confirmed DNA nicking and was accompanied by arrest of cell cycle progression.

**Conclusions:**

Taken together, compounds like MAL-A having pro-oxidant activity mediate their cytotoxicity in leukemic cells via induction of oxidative stress triggering a caspase dependent apoptosis.

## Introduction

In cancer cells, reactive oxygen species (ROS) are known to exert a paradoxical effect as they are critical both for cell survival and regulation of cell death [1]. Low concentrations of ROS can promote cancers by transforming normal cells through activation of transcription factors or inhibition of tumor suppressor genes, whereas on the other hand, elevated levels of ROS can also inhibit cancer progression via stimulation of pro-apoptotic signals leading to cell death [1]. Generally, tumor cells have higher levels of ROS than their normal counterparts owing to their increased metabolic activity, mitochondrial dysfunction, peroxisome activity, up-regulation of cellular receptor signalling pathways, oncogenic activity as also increased activity of pro-inflammatory cyclo-oxygenases and lipo-oxygenases ([2] and ref. therein, [3], [4]). However, this is countered by an effective anti-oxidant system that ensures redox homeostasis. Therefore, it may be extrapolated that anti-cancer compounds capable of inflicting additional oxidative stress may cause cell death. Indeed, there is emerging evidence that increased generation of ROS achievable by chemotherapy and/or radiotherapy can induce apoptosis in cancer cells [5], [6].

The fruit rind of the plant *Myristica malabarica* (Myristicaceae), popularly known as rampatri, Bombay mace, or false nutmeg is used as an exotic spice in various Indian cuisines. Its pharmacological activities range from hepatoprotective [7], anti-ulcerogenic [8] to anti-leishmanial [9]. Its phytoconstituents include diarylnonanoids of which malabaricone-C showed potent anti-oxidant [10] and anti-cancer activity which was attributed to its Cu(II)-dependent nuclease property [11].

In Leishmaniasis, a protozoan parasitic disease, the *Leishmania* parasites have an impaired anti-oxidant system ([12] and ref. therein) wherein triggering of oxidative stress has been demonstrated to be an effective chemotherapeutic modality ([13], [14] and ref. therein). Indeed, Miltefosine, that has anti-cancer [15] and anti-leishmanial activity [16] mediates its cytotoxicity via apoptosis. Therefore, considering that malabaricones have anti-leishmanial activity [9], it may be envisaged that it mediated its parasiticidal activity via its pro-oxidant property. Accordingly, we tested the anti-cancer potential of malabaricones and whether their ability to achieve cell death was via redox imbalance.

## Materials and Methods

### Materials

All chemicals if not otherwise stated were obtained from Sigma-Aldrich (St. Louis, Missouri, USA) except phenazine methosulphate (PMS), 5,5-dithiobis (2-nitrobenzoic acid, DTNB) and trichloroacetic acid (TCA) from Sisco Research Laboratories (Mumbai, India), MTS or 3-(4,5-dimethylthiazol-2-yl)-5-(3-carboxymethoxyphenyl)-2-(4-sulfophenyl)-2H-tetrazolium, inner salt (Promega, Madison, Wisconsin, USA), 5, 5′, 6, 6′-tetrachloro-1, 1′, 3, 3′-tetraethylbenzimidazolylcarbocyanine iodide (JC-1), fluo-4 acetoxymethyl (Fluo-4 AM), 5-chloromethylfluorescein diacetate (CMFDA), 5-(and-6)-chloromethyl-2′,7′-dichlorodihydrofluorescein diacetate (CMH_2_DCFDA) and Quick apoptotic DNA ladder detection kit (Invitrogen, Carlsbad, CA, USA), Z-Val-Ala-DL-Asp (methoxy)-fluoromethylketone (Z-VAD-FMK, BD Biosciences, San Jose, CA, USA), Caspase-3/CPP32, FLICE/caspase-8 and caspase-9 colorimetric assay kit (Biovision, Milpitas, CA, USA), Annexin-V FITC (ImmunoTools, Friesoythe, Germany), Cell Death Detection Kit (Roche, Penzberg, Germany) and 4,5 diaminofluorescein -2 diacetate (DAF-2DA, Cayman Chemicals, Ann Arbor, MI, USA), antibodies against poly (ADP-ribose) polymerase (PARP) and cytochrome *c* (Cell Signaling Technology, Inc. Beverly, MA, USA).

### Cell culture

Three human cell lines namely U937, a leukemic monocytic lymphoma, MOLT-3 an acute lymphoblastic leukemia and K562, a chronic myelogenous leukemia were used [17], [18]. All cell lines were maintained in RPMI 1640 medium (pH 7.4) supplemented with 10% heat inactivated fetal bovine serum (FBS), penicillin (50 units/ml) and streptomycin (50 µg/ml) at 37°C in a humidified incubator containing 5% CO_2_. The cells were sub-cultured every 48–72 h, inoculum being 5×10^5^/ml; cell viability (>95%) was confirmed by trypan blue exclusion.

### Isolation of peripheral blood mononuclear cells (PBMC)

Peripheral blood was carefully layered over Ficoll-Hypaque (1∶1, Histopaque-1077) and centrifuged (1700 rpm×30 minutes). The PBMC-rich interface was washed twice in phosphate buffered saline (PBS, 0.01 M, pH 7.4) and resuspended in RPMI-1640 medium supplemented with penicillin (50 U/ml), streptomycin (50 µg/ml) and 10% FBS. Cell viability was confirmed using trypan blue (>95%).

### In vitro evaluation of cytotoxic activity of Rampatri and derived malabaricones

The cytotoxic activity of a crude extract of Rampatri fruit rind and its purified fractions malabaricone-A (MAL-A), malabaricone-B (MAL-B), malabaricone-C (MAL-C) and malabaricone-D (MAL-D) was studied in U937 and MOLT-3 cell lines and cell viability measured using a modified MTS-PMS assay [19]. Briefly, cells (5×10^4^ cells/200 µl of RPMI 1640 medium/well) were incubated with the compounds (0–40 µg/ml) for 48 h following which cell viability was measured using MTS (2 mg/ml in phosphate buffered saline 0.02 M, pH 7.2, PBS) and PMS (0.92 mg/ml in PBS) that were added in the ratio of 10∶1 (20 µl per well). The plates were incubated for 3 h at 37°C in the dark and resultant optical densities measured at 490 nm in a microplate plate reader (OD_490_, Bio-Rad, Hercules, CA, USA). Accordingly, the specific absorbance that represented formazan production was calculated by subtraction of background absorbance from total absorbance; the mean % viability was calculated as follows:

Mean specific OD_490_ of treated cells ×100

Mean specific OD_490_ of untreated cells

Each experiment was repeated thrice in duplicates and after the data was plotted, the IC_50_ i.e. the concentration that inhibited 50% cell growth was enumerated by graphical extrapolation using Graph Pad Prism software (version 4, GraphPad Software Inc, San Diego, CA, USA). PBMC (5×10^4^/200 µl) was similarly incubated with MAL-A (0–40 µg/ml, 48 h) and cell viability measured as described above.

### Generation of intracellular reactive oxygen species (ROS) and reactive nitrogen species (RNS)

CM-H_2_DCFDA, a lipid soluble membrane permeable dye upon entering cells undergoes deacetylation by intracellular esterases and forms the more hydrophilic, non-fluorescent dye Dichlorodihydrofluorescein (DCFH_2_). This is subsequently oxidized by ROS to form a two-electron highly fluorescent oxidation product, Dichlorofluorescein (DCF); therefore, the fluorescence generated is directly proportional to the amount of ROS. Similarly, DAF-2DA is freely permeable, undergoes hydrolysis by cytosolic esterases, resulting in the formation of DAF-2 which in the presence of intracellular NO gets converted into an impermeable, highly fluorescent triazolofluorescein (DAF-2T). For both probes, fluorescence was acquired in a flow cytometer.

The effect of a near IC_50_ concentration of MAL-A (15 µg/ml) on generation of ROS (0–2 h) and RNS (0–1 h) was measured in cell lines (2.5×10^5^/ml). Following centrifugation (5000 rpm×5 minutes) and 2 washes, cells were resuspended in PBS, incubated for 30 minutes at 37°C with CM-H_2_DCFDA (5 µM) for measurement of ROS [17] or DAF-2DA (2 µM) for RNS [20].

### Flow cytometric determination of intracellular thiols

Non-protein thiols were measured as previously described [21]; briefly, U937 cells (2.5×10^5^/ml) were incubated with a near IC_50_ concentration of MAL-A (15 µg/ml) at 37°C for 0–6 h. Cells were then washed, resuspended in PBS and labelled with CMFDA (0.05 µM, 37°C, 15 minutes). CMFDA is a cell permeable, non-fluorescent dye that upon entering the cell, rapidly binds with non-protein thiols and becomes non-permeable; the simultaneous cleavage of the diacetate moiety by cellular esterases yields a fluorescent thioether whose fluorescence is acquired in a flow cytometer.

### Determination of glutathione peroxidase activity

The activity of glutathione peroxidase (GPx) was measured using DTNB as previously described [22]. Briefly, U937 protein lysate (50 µg in 100 µl of phosphate buffer, 0.4 M, pH 7.0) was added to a reaction mixture containing phosphate buffer (0.4 M, 90 µl), sodium azide (10 mM, 20 µl), reduced glutathione (4 mM, 40 µl, GSH), H_2_O_2_ (2.5 mM) and the final volume was made up to 400 µl by addition of double distilled water. After incubation at 37°C for 30 minutes, trichloroacetic acid (TCA, 10%, 100 µl) was added to stop the reaction, centrifuged (8000 rpm×5 minutes) and supernatant was collected. To the supernatant, phosphate buffer (0.3 M, 600 µl) and DTNB (200 µl, 4 mg/10 ml of 1% sodium citrate) was added and absorbances measured at 405 nm on a microplate reader; the concentration of unutilised GSH was determined from a standard curve using GSH (0–100 µg). The specific activity of GPx was calculated in IU/gm of protein. Total GSH was also measured using this protocol with sodium azide being replaced by β-NADPH (0.8 mM).

### Measurement of intracellular [Ca^2+^] using Fluo-4 AM

Changes in intracellular [Ca^2+^] were monitored using Fluo-4 AM as previously described [23]; briefly, U937 cells (2.5×10^5^/ml) were initially equilibrated at 37°C for 45 minutes with loading medium containing Fluo-4 AM (2.5 µM), pluronic acid F127 (0.02%) and sulfinpyrazone (0.25 mM) in RPMI 1640 medium. After de-esterification of Fluo-4 AM, cells were washed with RPMI 1640 medium containing sulfinpyrazone (0.25 mM, 37°C, 45 minutes). Following the addition of a near IC_50_ concentration of MAL-A (15 µg/ml), rapid kinetic measurement of fluorescence was performed thrice at an interval of 512.0 seconds by flow cytometry; Ca^2+^ ionophore (ionomycin 1 µM) and a chelating agent EGTA (3 mM, 15 minutes) served as the positive and negative control respectively.

### Analysis of mitochondrial transmembrane potential

The mitochondrial transmembrane electrochemical gradient (Δψ_m_) was measured using JC-1, [23]. JC-1, a cell permeable, cationic, lipophilic dye freely crosses the mitochondrial membrane and forms J-aggregates which fluoresce red; accordingly, viable cells with a normal mitochondrial membrane potential when stained with JC-1 exhibit a pronounced red fluorescence. Following an apoptotic stimulus, the resultant decrease in the mitochondrial membrane potential prevents JC-1 from entering the mitochondria and remains as monomers in the cytosol that emits a predominantly green fluorescence. Therefore, the ratio of J-aggregates/monomers serves as an effective indicator of the cellular mitochondrial transmembrane potential, allowing apoptotic cells to be easily distinguished from their non-apoptotic counterparts. Briefly, U937 cells (2.5×10^5^/ml) following incubation with MAL-A (0–15 µg/ml, 0–2 h, 37°C), were stained with JC-1 (7.5 µM in PBS, 10 minutes, 37°C). Cells were then acquired in a flow cytometer on the basis of quadrant plot to distinguish monomers from J-aggregates and analyzed using Cell Quest Pro software. To set the quadrants, cells were treated with H_2_O_2_ (20 mM, 37°C, 30 minutes), representative of cells with depolarized mitochondrial membrane potential.

### Measurement of cardiolipin oxidation

To determine the effect of MAL-A induced ROS upon cardiolipin peroxidation in mitochondria, 10-N-nonyl acridine orange (NAO) was used [17]. Briefly, U937 cells (2.5×10^5^/ml) after being treated with a near IC_50_ concentration of MAL-A (15 µg/ml, 0–12 h, 37°C) were washed with PBS, labeled with NAO (100 nM in methanol, 37°C, 10 minutes), acquired and analyzed in a flow cytometer.

### Measurement of Annexin V positivity

Double staining for Annexin V-FITC and propidium iodide (PI) was performed as previously described [23]. Translocation of phosphatidylserine from the inner aspect to the outer leaflet of the plasma membrane occurs during apoptosis which can be ascertained by exploiting the high binding affinity of Annexin V, a Ca^+2^ dependent phospholipid binding protein to phosphatidyl serine. To examine whether cell death occurred via apoptosis or necrosis, PI was used, which being a non permeable stain having affinity towards nucleic acids, selectively enters necrotic or late apoptotic cells. Therefore, co-staining of Annexin V and PI helps discriminate between live cells (PI and Annexin V negative), cells in early apoptosis (Annexin V positive, PI negative), cells undergoing late apoptosis (both Annexin V and PI positive) or necrotic cells (PI positive, Annexin V negative).

Briefly, U937 cells (2.5×10^5^/ml) were incubated with a near IC_50_ concentration of MAL-A (15 µg/ml) at 37°C, 5% CO_2_ for 0–24 h. After two washes, cells were resuspended in Annexin V binding buffer (10 mM HEPES/NaOH, pH 7.4, 140 mM NaCl, 2.5 mM CaCl_2_) and Annexin V–FITC was added according to the manufacturers' instructions. The cells were incubated for 30 minutes in the dark at 37°C and just prior to acquisition, PI (0.1 µg/ml) was added and cells were acquired in a flow cytometer.

### Determination of caspase activity

Activity of caspases-8, 9 and 3 was detected in cell lysates (100 µg protein in 50 µl lysis buffer) as per the manufacturer's instructions. Briefly, U937 cells (2.5×10^5^/ml) after incubation with MAL-A (15 µg/ml, 18 h at 37°C) was washed twice with ice cold PBS, cell lysates prepared and protein concentration estimated. Lysates were combined with 50 µl of 2× reaction buffer (containing 10 mM DTT), caspase 3 substrate DEVD-pNA (4 mM, 5 µl) or caspase 8 substrate IETD-pNA (4 mM, 5 µl) or caspase 9 substrate LEHD-pNA (4 mM, 5 µl) following incubation at 37°C for 0–3 h; the release of chromophore para nitroanilide (pNA) was quantified by measuring absorbances at 405 nm every 30 minutes for 3 h. To establish whether MAL-A induced death was caspase independent or not, U937 cells (5×10^4^ in 200 µl/well) were preincubated with a pan caspase inhibitor Z-VAD-FMK (20 µM, 1 h) followed by 48 h co-incubation with MAL-A and cell viability evaluated by the MTS-PMS assay as described above.

### Immunoblotting

Cells were resuspended in cell lysis buffer [Tris-HCl, (50 mM, pH-7.5), EGTA (50 mM), β-mercaptoethanol (50 mM) and protease inhibitors, leupeptin (0.33 mM), phenylmethylsulfonyl fluoride (0.2 mM), antipain (0.35 mM), chymostatin (0.24 mg/ml), pepstatin (0.35 mM) and aprotinin (4.8 units/ml)] and after sonication, centrifuged (12,400 rpm×2 minutes) and protein concentration measured. Samples were boiled with sample buffer for 5 minutes and stored at −20°C. Lysates (75 µg protein per lane) were resolved by 10% SDS-PAGE and transferred onto nitrocellulose membranes. After blocking the non specific binding sites with 5% non fat dried milk in Tris buffered saline (50 mM, pH 7.4, TBS) for 2 h, membranes were incubated overnight at 4°C with antibodies against poly (ADP-ribose) polymerase (PARP, 1∶500) and cytochrome *c* (1∶1000) in TBS containing 5% BSA. Membranes were washed thrice with TBS containing 0.1% Tween 20 and binding detected with HRP conjugated secondary antibody (anti rabbit IgG, 1∶2000, 2 h, room temperature in TBS containing 2% BSA), the bands being visualized using diaminobenzidine (10 ml of 100 mM Tris-HCl, pH 7.5 containing 200 µl of 40 mg/ml diaminobenzidine in water and 30 µl of 3% stabilized H_2_O_2_) and analyzed using G-BOX gel doc apparatus (Syngene, Cambridge, UK; [24]).

### In situ detection of DNA fragmentation by terminal deoxynucleotidyl Transferase mediated dUTP nick end labeling (TUNEL)

*In situ* detection of DNA fragments was measured by terminal deoxyribonucleotide transferase (TdT) mediated dUTP nick end labeling (TUNEL) according to the manufacturer's instructions. Briefly, U937 cells (2.5×10^5^/ml) were treated with a near IC_50_ concentration of MAL-A (15 µg/ml) for 24 and 48 h at 37°C; cells were washed, fixed with paraformaldehyde (2% in PBS, pH 7.4) and after being kept on ice for 1 h, cells (2×10^5^) were centrifuged (5000 rpm×5 minutes), resuspended in PBS (10 µl), and spotted on glass slides. The air dried slides were washed with PBS, placed on ice and permeabilized with freshly prepared, chilled Na-Citrate (0.1%, w/v) in Triton X-100 solution (0.1%, v/v) for 2 minutes. Cells were washed twice with PBS following which a reaction mixture (25 µl) containing enzyme (TdT) and nucleotide mixture was added. The slides were then incubated in a humidified chamber at 37°C for 1 h, washed with PBS and convertor POD (anti fluorescein antibody conjugated with horse – radish peroxidase, 25 µl) was added and incubated for 30 minutes at 37°C. Finally, the substrate diaminobenzidine (25 µl) was added, slides were kept at 4°C for 10 minutes, washed with deionised water and observed microscopically under oil immersion objective (1000× magnification). At least 20 randomly selected microscopic fields were examined. Images were taken using a digital compact camera with a 4× zoom (Olympus, Singapore, CAMEDIA, C-7070) and modified using Adobe Photoshop 7.0 (Adobe Systems Inc., Mountain View, CA, USA).

### Measurement of DNA laddering

To determine DNA laddering, total cellular DNA was isolated from U937 cells (2.5×10^5^/ml) previously treated with a near IC_50_ concentration of MAL-A (15 µg/ml, 48 h) according to manufacturer's instructions and analyzed by gel electrophoresis (1.5% agarose gel) and visualized on a G-BOX gel doc apparatus (Syngene, Cambridge, UK; [23]).

### Measurement of nuclear chromatin condensation

Apoptotic cells were also characterized by nuclear condensation of chromatin and/or nuclear fragmentation [25]. Briefly, U937 cells (2.5×10^5^/ml) incubated with a near IC_50_ concentration of MAL-A (15 µg/ml, 24 h), were washed with ice cold PBS, stained with DAPI (2.5 µg/ml, 20 minutes) and mounted on poly L-lysine coated slides for analysis on a laser scanning confocal microscope (Leica TCS SP2 system, Leica microsystems, Heidelberg, Germany, 100×); at least 20 randomly selected microscopic fields were observed per sample.

### Cell cycle analysis

U937 cells (2.5×10^5^/ml) treated with MAL-A (15 µg/ml, 0–24 h, 37°C/5% CO_2_) were fixed in chilled ethanol (70%) and kept at 4°C until analysis. Prior to analysis, cells were washed in PBS containing 2% FBS and the resultant pellet resuspended in DNase-free RNase (200 µg/ml, 0.5 ml) for 2 h at 37°C; cells were then stained with PI (40 µg/ml) and acquired on a flow cytometer [23]. The data were analysed using Cell Quest Pro software and expressed as % of cells in each phase of cell cycle.

### Flow cytometry

U937 cells (5×10^5^) from different experimental groups were monitored for their intracellular fluorescence on a flow cytometer (FACS Calibur, Becton Dickinson, San Jose, CA, USA) equipped with an argon-ion laser (15 mW) tuned to 488 nm. The fluorescence of DCF, glutathione sulphomethyl fluorescein (GSMF) and DAF-2T were collected in the FL1 channel, equipped with a 530/30 nm band pass filter while PI was measured in the FL2 channel having a 585/42 nm band pass filter and NAO in the FL3 channel having a 682/33 nm band pass filter. Fluorescence was acquired in the log mode and expressed as geometrical mean fluorescence channel (GMFC) or the average or central tendency of fluorescence of analyzed particles. Acquisition was performed on 10,000 gated events. The data were analyzed using either histogram or quadrant plot with CellQuest Pro software (BD Biosciences, San Jose, CA, USA).

### Statistical Analysis

Each experiment was performed at least thrice in duplicates and results expressed as mean ± SEM/SD. Statistical analysis was evaluated by Students t-test and one way ANOVA followed by Tukey multiple comparison test (wherever applicable), using Graph Pad Prism software, version 4 (GraphPad Software Inc, San Diego, CA, USA); p<0.05 was considered as statistically significant.

## Results

### Cytotoxicity of Rampatri and its derived compounds

**Figure 1:**
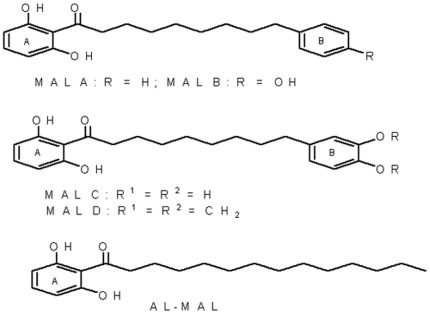
The chemical structure of malabaricone derivatives (MAL-A, MAL-B, MAL-C, MAL-D and AL-MAL) isolated from the methanolic extract of rampatri (*Myristica malabarica*).

Diarylnonanoids isolated from a methanolic extract of the fruit rind of *Myristica malabarica* namely malabaricone-A (MAL-A), malabaricone-B (MAL-B), malabaricone-C (MAL-C) and malabaricone-D, MAL-D (Figure 1) [10] were tested for their cytotoxic potential in leukemic cell lines, U937 and MOLT-3. The crude extract and the malabaricones, except MAL-C (IC_50_>50 µg/ml), showed an IC_50_ (mean ± SEM) ranging from 12.7±0.46 to 24.5±0.44 µg/ml in U937 and 12.3±1.67 to 20.4±1.72 µg/ml in MOLT-3 cell lines (Table 1); DMSO (0.2%), representative of the highest concentration present in malabaricones (40 µg/ml) showed no effect on cell viability, confirming its biological inertness. As MAL-A and MAL-D were comparable as regards their cytotoxicity, we proceeded to study MAL-A as a representative compound.

**Table 1:** Cytotoxicity of crude extract of Rampatri and its derived compounds.

Compounds	U937	MOLT-3
	*(µg/ml, Mean ± SEM)	*(µg/ml, Mean ± SEM)
Crude	24.5±0.44	12.3±1.67
MAL-A	12.7±0.46	16.9±1.64
MAL-B	19.1±0.16	20.4±1.72
MAL-C	>50	>50
MAL-D	13.3±0.25	19.3±2.35

* U937 and MOLT-3 cells (5×10^4^/200 µl) were treated with a crude extract of Rampatri and its derived compounds (0–40 µg/ml) for 48 h, and cell viability was measured by MTS-PMS assay as described in Materials and methods; each data is the IC_50_ (Mean ± SEM) derived from at least three experiments in duplicate.

### MAL-A caused a redox imbalance in U937 cells

**Figure 2:**
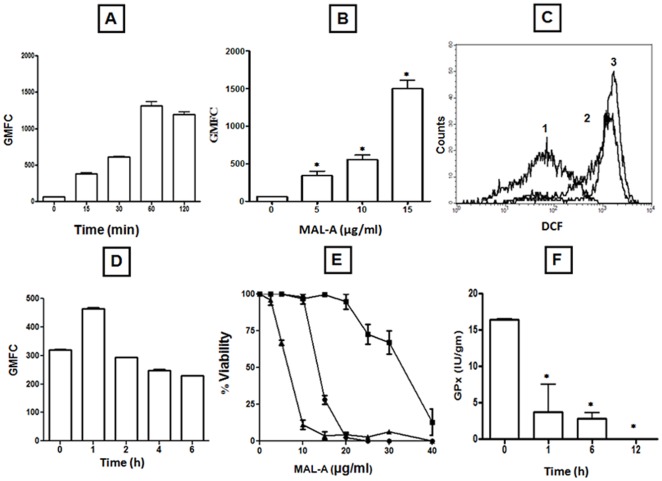
Modulation of oxidant status of U937 cells by MAL-A. (A) Time dependent effect of MAL-A on generation of ROS. Cells (5×10^5^) were incubated with MAL-A (15 µg/ml) for 0–120 minutes, labelled with CM-H_2_DCFDA (0.5 µM) and analyzed for fluorescence of DCF as described in Materials and methods. Data are expressed as mean GMFC ± SEM of at least three experiments in duplicate. (B) Concentration dependent effect of MAL-A on generation of ROS. Cells (5×10^5^) were incubated with MAL-A (0–15 µg/ml) for 1 h, labelled with CM-H_2_DCFDA (0.5 µM) and analyzed for fluorescence of DCF as described in Materials and methods. Data are expressed as the mean GMFC ± SEM of at least three experiments in duplicate. *p<0.0001 as compared to controls. (C) A representative histogram profile of DCF fluorescence. U937 cells (5×10^5^/ml, 1, unstained) were incubated in the presence of MAL-A at concentrations of 10 (2) and 15 µg/ml (3) for 1 h followed by labeling with CM-H_2_DCFDA (0.5 µM) and GMFC measured as described in Materials and methods. (D) Effect of MAL-A on levels of non-protein thiols. Cells (5×10^5^) were incubated with MAL-A (15 µg/ml) for 0–6 h, labelled with CMFDA (0.05 µM) and analyzed for fluorescence of CMF as described in Materials and methods. Data are expressed as mean GMFC ± SEM of at least three experiments in duplicate. (E) Effect of NAC and BSO on survival of U937. Cells (5×10^4^/200 µl/well) were incubated with MAL-A (0–40 µg/ml, •) along with NAC (2.5 mM, ▪) or BSO (5 mM, ▴) for 48 h and cell viability was measured by the MTS-PMS assay as described in Materials and methods. Each point corresponds to the mean ± SEM of at least three experiments in duplicate. (F) Effect of MAL-A on glutathione peroxidase activity (GPx). U937 cells were incubated with MAL-A (15 µg/ml) for 0–12 h followed by cell lysis and GPx activity was determined as described in Materials and methods; Data are expressed in IU/gm of protein mean ± SEM of at least three experiments in duplicate. *p<0.0001 as compared to controls.

Leukemic cells have been reported to have an inherently higher level of ROS in comparison with normal lymphocytes and could be expected to have greater sensitivity to oxidative assault ([2] and references therein, [3]). Accordingly, we measured the levels of ROS in three leukemic cell lines in the absence and presence of MAL-A. The basal ROS generated in all three cell lines U937, MOLT3 and K562 in terms of GMFC was 60.01±0.91, 54.14±2.48 and 41.90±5.04 respectively, whereas in PBMC, the GMFC was lower being 34.60±5.05. U937 cells when incubated with a near IC_50_ concentration of MAL-A (15 µg/ml, 0–2 h) showed a time dependent increase in generation of ROS, maximum being at 1 h (Figure 2A). A concentration dependent response was also observed, as MAL-A at the highest concentration of 15 µg/ml caused a 24.92 fold increase in fluorescenceas compared to baseline, GMFC being 1496.00±169.10 (p<0.0001, Figures 2B and C). In MOLT3 and K562 cell lines, MAL-A (15 µg/ml, 1 h) the generation of ROS induced was 5.86 (317.58±7.23, p<0.05) and 9.65 (404.42±24.27, p<0.05) fold higher respectively than their basal levels. However, in PBMC, the basal levels of ROS increased marginally from baseline, GMFC being 57.53±9.10. There was no change in cell viability as measured by PI exclusion (data not shown). The auto-fluorescence generated by MAL-A was minimal, indicating that the observed increase in fluorescence was attributable to its ability to generate ROS.

As nitric oxide (NO) is an important signaling and effector molecule that along with ROS can be cytotoxic [26], we examined the effect of MAL-A on levels of NO using DAF-2DA. U937 cells, when incubated with a near IC_50_ concentration of MAL-A (15 µg/ml, 0–1 h) showed a 1.7 fold increase in generation of NO from basal level, maximum being at 30 minutes, GMFC being 319.30±4.30 vs.186.10±1.90.

As a redox imbalance can also be generated via depletion of the anti-oxidant component, we examined the effect of MAL-A on levels of non-protein thiols using CMFDA. Treatment with a near IC_50_ concentration of MAL-A (15 µg/ml) decreased levels of reduced non-protein thiols by 22.5% that translated into the GMFC (mean ± SEM) decreasing from a baseline fluorescence of 319.80±1.90 to 247.90±3.70 at 6 h (Figure 2D).

To establish whether MAL-A caused impairment of the anti-oxidant component and thereby contributed towards the cytotoxicity of MAL-A, cells were pre-incubated with a non toxic concentration of buthionine sulfoximine (BSO, 5 mM, 1 h), an established GSH depletor, followed by MAL-A (0–40 µg/ml, 48 h). In the presence of BSO, the IC_50_ (mean ± SEM) of MAL-A decreased 2.01 fold from 12.7±0.46 µg/ml to 6.40±0.20 µg/ml substantiating that if a redox imbalance is generated, it enhances the cytotoxicity of MAL-A (Figure 2E). Additionally, MAL-A (15 µg/ml) in U937 cells significantly decreased the specific activity of GPx (mean ± SEM) from 16.38±0.06 IU/g protein to 3.70±1.41 and 2.70±0.30 IU/g protein at 1 and 6 h respectively, while at 12 h, no measurable activity was obtained (Figure 2F); the total GSH remained unchanged (data not shown).

To corroborate that MAL-A induced oxidative stress (Figures 2B–D) is essential for mediating its cytotoxicity, U937 cells were co-incubated with MAL-A and a non toxic concentration of N-acetyl-L-cysteine (NAC, 2.5 mM), an established anti-oxidant. With the addition of NAC, the cytotoxic effect of MAL-A was attenuated, as its IC_50_ (mean ± SEM) increased 2.5 fold from 12.70±0.46 µg/ml to 32.85±0.55 µg/ml (Figure 2E) revalidating that induction of oxidative burst and subsequent redox imbalance is a key factor for mediating the cytotoxicity of MAL-A.

### MAL-A increased cytosolic [Ca^2+^]

**Figure 3:**
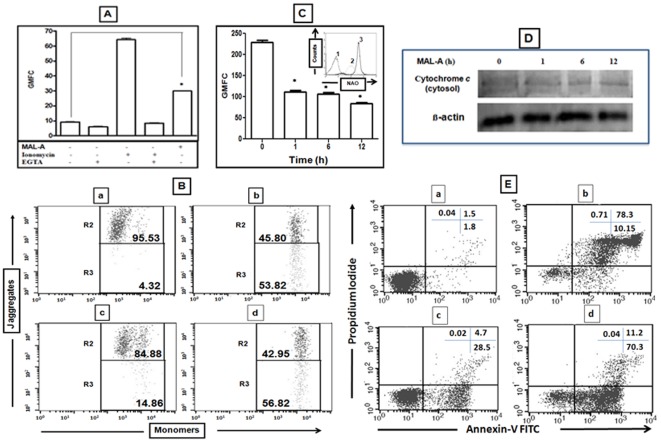
Induction of apoptosis by MAL-A. (A) Changes in [Ca^2+^] levels. U937 cells (5×10^5^) were pre-incubated with Fluo-4AM (2.5 µM, 45 minutes, 37°C) followed by rapid kinetic measurement of fluorescence in the presence of MAL-A (15 µg/ml). Ionomycin (1 µM) served as a positive control along with EGTA (3 mM, 15 minutes) as a negative control as described in Materials and methods. Data are expressed as mean ± SEM of at least three experiments in duplicate. *p<0.0001 as compared to controls. (B) Effect of MAL-A on mitochondrial transmembrane potential. U937 Cells (5×10^5^, a) were incubated with H_2_O_2_ (20 mM, 30 minutes as positive control, b), MAL-A (10 µg/ml, c) or 15 µg/ml (d) and probed with JC-1 as described in Materials and methods. The encompassed population of R2 represents J-aggregates whereas R3 represents monomers. The data is a representative profile of at least three experiments in duplicate. (C) Effect of MAL-A on peroxidation of mitochondrial cardiolipin. U937 cells (5×10^5^) were incubated with MAL-A (15 µg/ml) for 1, 6 and 12 h and then labelled with NAO (100 nM) after which they were analyzed for fluorescence as described in Materials and methods. Data are expressed as mean GMFC ± SEM of at least three experiments in duplicate. *p<0.0001 as compared to controls. Inset: A representative profile of NAO fluorescence: U937 cells (5×10^5^/ml, 1, unstained) were incubated in the presence (2) or absence (3) of MAL-A (15 µg/ml) for 12 h at 37°C followed by labeling with NAO (100 nM) and GMFC measured as described in Materials and methods. (D) Effect of MAL-A on release of cytochrome *c* into cytosol. U937 cells were treated with MAL-A (15 µg/ml) for 1, 6 and 12 h; mitochondrial and cytosolic fractions were separated and subjected to western blotting with anti cytochrome *c* antibody along with β-actin used as loading control for the cytosolic fraction as described in Materials and methods. The figure is a representative profile of at least three experiments. (E) Externalization of phosphatidyl serine by MAL-A. U937 cells (5×10^5^, a) were incubated with an inducer of apoptosis BD68 (0.5 µM, 8 h, b), MAL-A; 15 µg/ml for 2 h (c) or 4 h (d), co-stained with PI and Annexin-V FITC followed by analysis by flow cytometry as described in Materials and methods. The figure is a representative profile of at least three experiments.

Changes in the redox potential can cause alterations in cytosolic [Ca^2+^] which then induces mitochondrial dysfunction [27]. The addition of Ionomycin, a potent, Ca^2+^ ionophore, triggered an increase in intracellular [Ca^2+^] which decreased in the presence of EGTA (a Ca^2+^ chelator), confirming assay specificity. Treatment of U937 cells with a near IC_50_ concentration of MAL-A (15 µg/ml, 0–512 seconds) triggered a rapid elevation of intracellular [Ca^2+^] causing a 3.3 fold enhancement of fluorescence (8.90±0.19 vs. 29.71±0.20, p<0.0001, Figure 3A) and thereafter remained unaltered.

### MAL-A induced depolarization of mitochondrial transmembrane potential

The loss of mitochondrial membrane potential is a characteristic feature of apoptosis which can be measured using JC-1, as the ratio of J-aggregates/monomers serves as an effective indicator of the cellular mitochondrial transmembrane potential; this allows apoptotic cells to be easily distinguished from their non-apoptotic counterparts [28]. In control U937 cells, the red/green fluorescence ratio was 22.11 which following the addition of MAL-A (10 and 15 µg/ml, 1 h) dramatically decreased to 5.71 and 0.75 respectively (data not shown).

JC-1 fluorescence was also measured by estimating the % gated population i.e. mean ± SEM in R2 and R3, wherein R2 represented the healthy, non apoptotic J-aggregates while R3 represented the apoptotic, monomeric cell population (Figure 3B). This gating was set following addition of H_2_O_2_ (20 mM, 30 minutes) wherein the mean ± SEM in R2 and R3 was 45.80±6.32% and 41.20±6.46% respectively. In healthy cells, the R2 and R3% positivity was 95.61±0.44% and 4.29±0.42% respectively while treatment with MAL-A (10 and 15 µg/ml) progressively increased the R3 gated population to 48.92±10.40% and 72.47±15.60% respectively (Figure 3B), indicating that MAL-A rapidly induced depolarization of mitochondrial membrane potential, an early indicator of apoptosis.

### MAL-A caused peroxidation of cardiolipin

10-N-nonyl-acridine orange (NAO) a fluorescent dye has a high binding affinity specifically for mitochondrial cardiolipin; therefore, following peroxidation of mitochondrial cardiolipin, the resultant decrease in fluorescence of NAO indicates enhanced peroxidation of cardiolipin [29]. As oxidative stress causes peroxidation of mitochondrial cardiolipin, we evaluated the effect of MAL-A upon NAO fluorescence. U937 cells when incubated with a near IC_50_ concentration of MAL-A (15 µg/ml) demonstrated a time dependent decrease in fluorescence as the mean ± SEM of GMFC progressively decreased from 228.50±5.20 (untreated cells) to 111.5±3.40, 106.50±4.00 and 83.88±2.90 at 1, 6 and 12 h respectively (p<0.0001, Figure 3C).

### MAL-A induced release of cytochrome c

As cytochrome *c* is bound to the inner membrane of mitochondria by anionic phospholipids e.g. cardiolipin, it is known that mitochondrial membrane depolarization and peroxidation of cardiolipin causes cytochrome *c* to be released to the cytosol, which then activates the initial events of apoptosis. In the absence of MAL-A (0 h, lane 1, Figure 3D), absence of a band was evidence of mitochondrial intactness and as we have studied the status of cytochrome c in the cytosol, β-actin served as the loading control. MAL-A effectively induced depolarization of mitochondrial membrane potential (Figure 3B) and peroxidation of cardiolipin (Figure 3C), that was evident from 1 h onwards and led to release of cytochrome *c* to cytosol which too was evident from 1 h (Figure 3D).

### MAL-A increased externalization of phosphatidylserine

In U937 cells, the basal binding of Annexin V was 2.67±1.43% which MAL-A at 2 and 4 h, increased to 33.21±0.05% and 82.2±0.17% respectively, indicating that MAL-A induced apoptosis in majority of the population; the percentage of PI-positive cells at baseline was minimal, and importantly was unchanged at 2 and 4 h being 0.04±0.07% and 0.04±0.08% respectively (Figure 3E). Taken together, MAL-A caused externalization of phosphatidyl serine to a degree comparable with BD68, an inducer of apoptosis [30].

### MAL-A caused apoptosis via a caspase dependent pathway

**Figure 4:**
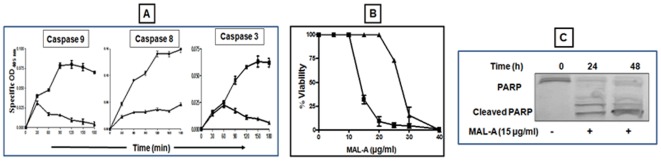
Effect of MAL-A on caspase activity and cleavage of PARP. (A) Lysates of U937 cells (▴) following treatment with MAL-A (▪) were used to study the activity of caspase 9, 8 and 3 as described in Materials and methods. Each point corresponds to the mean ± SD of at least three experiments in duplicate. (B) Effect of Z-VAD FMK on cell viability. U937 cells (5×10^4^/200 µl/well) were incubated with MAL-A (0–40 µg/ml, ▪) and Z-VAD FMK (20 µM, ▴) for 48 h and cell viability measured by the MTS-PMS assay as described in Materials and methods. Each point corresponds to the mean ± SD of at least three experiments in duplicate. (C) MAL-A induced PARP cleavage. U937 cells were treated with MAL-A (15 µg/ml, 24 h and 48 h) as described in Materials and methods. The figure is a representative profile of at least three experiments.

As caspases are effector molecules of the extrinsic and intrinsic apoptotic pathways, their activity was examined in MAL-A treated cells. Lysates were prepared from cells treated with MAL-A for 18 h based on a dose response pilot study wherein maximal caspase activity was observed (data not shown). An exponential increase in the activity of all three caspases was observed up to 2 h after which the activity plateaued. The increased activation of caspase 8 (∼135 fold), 9 (∼75 fold) and 3 (∼57 fold, Figure 4A), collectively indicated the potential of MAL-A to induce a marked degree of caspase activation. To confirm the role of caspases in MAL-A induced cytotoxicity, U937 cells were co-incubated for 48 h with MAL-A (0–40 µg/ml), in the absence/presence of a non toxic concentration of Z-VAD-FMK (20 µM), a pan caspase inhibitor and cell viability was measured. The addition of Z-VAD-FMK attenuated MAL-A induced cytotoxicity, as its IC_50_ increased to 26.1 from 13.2 µg/ml validating that induction of apoptosis was a caspase-dependent phenomenon (Figure 4B).

### MAL-A induced cleavage of poly (ADP) ribose polymerase (PARP)

PARP, a DNA repair enzyme serves as a substrate for active effector caspase 3 and therefore when cells undergo apoptosis and the caspase cascade is activated, activated effector caspase 3 causes cleavage of PARP, resulting in abrogation of the DNA repair machinery, thereby enhancing cell death. As MAL-A activated the caspase cascade in U937 cells it also effectively cleaved PARP (Figure 4C).

### MAL-A induced nuclear chromatin condensation

**Figure 5:**
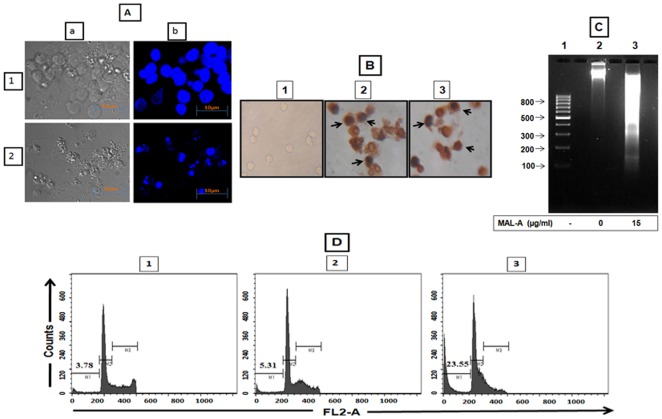
Apoptosis related nuclear events induced by MAL-A. (A) Nuclear chromatin condensation. U937 cells (5×10^5^, 1) were treated with MAL-A (15 µg/ml, 2), labelled with DAPI, fluorescence was analyzed by laser scanning confocal microscopy as described in Materials and methods. a and b represent phase contrast and fluorescence panel respectively and the figure is a representative profile of at least three experiments. Bar size is 10 µm. (B) TUNEL positivity. U937 cells (1) were treated with MAL-A (15 µg/ml, 24 h, 2) or H_2_O_2_ (1 mM, 24 h as positive control, 3) and processed for *in situ* TUNEL staining as described in Materials and methods. The arrows represent incorporation of TdT-labeled nuclei. The figure is a representative profile of at least three experiments. (C) DNA fragmentation. U937 cells (2) treated with MAL-A (15 µg/ml, 48 h, 3) and processed for DNA isolation as described in Materials and Methods. The figure is a representative profile of at least three experiments, lane 1: 100 bp DNA ladder. (D) Effect of MAL-A on cell cycle progression. U937 cells (1) were treated with MAL-A (15 µg/ml, for 6 h, 2) and 24 h (3) following which they were processed for cell cycle analysis as described in Materials and methods. M1, M2 and M3 represented % of cells in sub G_0_/G_1_, G_0_/G_1_ and S+G_2_/M stage of cell cycle respectively. The figure is a representative profile of at least three experiments.

Chromatin condensation is a feature of apoptotic cells; using DAPI, a nucleic acid binding dye, U937 cells treated with MAL-A (15 µg/ml, 24 h) showed nuclear chromatin condensation (Figure 5A), further evidence of its apoptotic potential.

### MAL-A induced DNA nicking and oligonucleosomal DNA fragmentation

As single stranded nuclear DNA nicking is one of the features of apoptosis, the *in situ* TUNEL staining was performed on U937 cells treated with MAL-A (15 µg/ml, 24 h) based on its proven ability to detect single strand breaks. MAL-A (15 µg/ml, 24 h), caused brown deposits, representative of incorporated TdT catalysed-labelling of nuclei, as was with H_2_O_2_ (1 mM, 24 h) that served as a positive control (Figure 5B).

Another hallmark of apoptotic cell death is internucleosomal DNA digestion by endogenous nucleases yielding a characteristic laddering pattern. Accordingly, oligonucleosomal DNA fragmentation following treatment of U937 cells with MAL-A (15 µg/ml, 48 h) was studied, wherein a degree of smearing was evident (Figure 5C).

### MAL-A increased the sub G_0_/G_1_ population

Flow cytometric analysis helped to quantify the percentage of U937 cells in different phases of the cell cycle, the amount of bound PI representing DNA content. Accordingly, DNA fragmentation that occurs in apoptotic cells translates into a fluorescence intensity lower than G_0_/G_1_ cells, which is considered as the sub G_0_/G_1_ phase. A near IC_50_ concentration of MAL-A (15 µg/ml), increased the proportion of cells in the sub G_0_/G_1_ phase, mean ± SD of % gated cells at 6 and 24 h being 5.52±0.30% and 22.02±2.15% respectively, whereas in controls, it remained at 2.19±1.40% (Figure 5D, Table 2). Taken together, the progressive increase in proportion of cells in the sub G_0_/G_1_ phase corroborated that MAL-A induced DNA degradation in U937 cells.

**Table 2:** Effect of MAL-A on cell cycle progression of U937 cells.

Cells	*M1 (sub G_0_/G_1_)	*M2 (G_0_/G_1_)	*M3 (S+G_2_/M)
**Control**	2.19±1.46	58.73±5.31	43.20±3.69
**MAL-A (6 h)**	5.52±0.30	58.71±6.52	35.60±6.64
**MAL-A (24 h)**	22.02±2.15	56.14±6.17	21.70±8.82

* U937 cells (1×10^6^) were treated with MAL-A (15 µg/ml) for 6 and 24 h and processed for cell cycle analysis as described in Materials and methods. Values are expressed as percentages (%), Mean ± SD and the data is a representative profile of at least three experiments in duplicate. M1, M2 and M3 represents % of cells in sub G_0_/G_1_, G_0_/G_1_ and S+G_2_/M stage of the cell cycle respectively.

## Discussion

Natural compounds have shown promising outcomes in cancer therapy and provided many lead structures, which have subsequently been used to develop compounds with enhanced biological properties [31]. There is mounting evidence to suggest that enhanced generation of ROS plays an important role in cancer biology. It has been recognized to play a ‘two-faced’ role displaying both deleterious and beneficial effects ([32] and ref. therein). ROS can act as secondary messengers in intracellular signaling cascades which help to induce and sustain the oncogenic phenotype of cancer cells ([33] and ref. therein). In cancer cells, the basal levels of ROS are higher and is often accompanied by an enhanced anti-oxidant system vis a vis their normal counterparts [34 and ref. therein]. However, if an oxidative assault beyond a critical threshold is mounted, it actually leads to an imbalance in the redox homeostasis and can translate into apoptosis ([35] and ref. therein).

Rampatri and its phytoconstituents has been reported to have anti-oxidant [10], anti-ulcerogenic [8], hepatoprotective [7], anti-leishmanial effects [9] and anti-cancer effects [11]. Among the phytoconstituents, MAL-A, MAL-B and MAL-D demonstrated comparable cytotoxicity in two leukemia cell lines (U937 and MOLT 3) which possibly accounts for the potent cytotoxicity of the crude extract (Table 1). Importantly, MAL-C which showed effective anti-ulcer and anti-oxidant activity showed minimal anti-cancer activity in leukemic cells (Table 1). Interestingly, MAL-C when supplemented with Cu in a MCF-7 breast cancer cell line, was cytotoxic, while MAL-A and MAL-D were ineffective, suggesting that Malabaricones have variable effect in solid tumors vs. lymphatic cells [11].

Earlier studies revealed that diarylnonanoids isolated from the methanol extract of rampatri exhibited anti-oxidant [10] and anti-leishmanial activity [9]. Structurally, all the malabaricones possess a 2-acylresorcinol moiety (Ring A), and differ in terms of substitution in their respective aromatic B rings (Figure 1). With regard to their B ring, the four compounds (MAL A–D) differed in respect to the presence of oxygenated functionalities. Of these, the B ring of MAL-A is devoid of any group, while in MAL-C and MAL-B, the B ring contains a catechol and a phenolic moiety respectively. However, in MAL-D, the two adjacent oxygenated functions present in the B ring is a methylenedioxy group and thereby the -OH groups are completely blocked. As the radical scavenging potency of substituted phenols is governed by the electron-donating effect of the substituents, MAL-C is a strong anti-oxidant as the activity of a strong electron-donating group, such as the hydroxyl group, at its ortho and para positions is much higher, MAL-B also shows some anti-oxidant property but relatively less than MAL-C.

As it has been proposed that the malabaricone-induced apoptotic death in U937 cells is governed by the augmented cellular oxidative status, it follows that MAL-A and MAL-D should show highest anti-cancer activity, followed by MAL-B, while MAL-C should have the lowest cytotoxicity. Indeed, our results were consistent with this logic as corroborated by the IC_50_ data (Table 1). A malabaricone congener (designated as AL-MAL) having an aliphatic side chain in place of the aromatic B ring was completely inactive in all these cell lines (data not shown) implying that the aromatic B ring may play a secondary role in their anti-cancer property (Figure 1).

As leukemic cells have been reported to have a higher basal content of ROS and are more sensitive to pro-oxidants as compared to their normal counterparts i.e. PBMC [3], we tested the effectiveness of malabaricones to generate ROS in leukemic cell lines (U937, MOLT3 and K562). Exposure of U937 cells to an IC_50_ concentration of MAL-A dramatically enhanced generation of ROS (Figures 2A–C), as also in other leukemic cell lines, MOLT3 and K562 corroborating with previous reports [2 and ref. therein]. Among these three leukemic cells, the generation of ROS was maximal in U937 when treated with MAL-A.

The effects of NO are modulated via direct and indirect interactions that can be cell-type specific [26] as for example in U937, HL-60, HeLa, Jurkat and PC-12 cell lines, NO induced apoptosis. However, in B lymphocytes, GT39, L929 and PC12, rat lung epithelial (RLE) cells, it conversely inhibited apoptosis [26]. In U937 cells, NO is reported to be an inducer of apoptosis at lower doses and necrotic at higher doses [36]. As MAL-A increased the generation of NO, it contributed towards the redox imbalance, necessary to induce apoptosis. However, as MAL-A only marginally depleted levels of GSH (Figure 2D), it suggested its potential to impair the enzymatic mechanisms regulating levels of reduced glutathione were possibly altered. Accordingly, the activity of glutathione peroxidase (GPx) was studied which catalyses oxidation of GSH to GSSG by utilizing H_2_O_2_; in turn glutathione reductase catalyzes the conversion of GSSG to GSH and thus protects the cell from oxidative stress [37]. Taken together, the pronounced down regulation of GPx activity by 77.3, 84.4 and 100% at 1, 6 and 12 h respectively, validated that in leukemic cells, MAL-A caused perturbation of the redox homeostasis via enhanced generation of ROS and depletion of the anti-oxidant component (Figure 2F).

To confirm the role of ROS in mediating MAL-A induced cell death, a thiol specific anti-oxidant, N-acetyl-L-cysteine (NAC) was used, assuming that if it attenuated the cytotoxicity of MAL-A [38], it would confirm the critical contribution of ROS. Indeed, pre-treatment with NAC scavenged intracellular ROS causing the IC_50_ to increase 2.5 fold (Figure 2E), proving that oxidative damage is a key player. This is in agreement with previous reports that NAC decreases the activity of ROS-dependent anti-cancer agents such as arsenic trioxide and sulforaphane [39]. Furthermore, a redox imbalance triggered by depleting GSH using a non-toxic concentration of an established GSH depletor, buthionine sulfoximine (BSO, 5 mM), enhanced the cytotoxicity of MAL-A (Figure 2E), corroborating that redox imbalance was the key pathway through which MAL-A caused cell death.

Ca^2+^ is a universal signaling molecule regulating several cellular functions and is one of the key elements in the apoptotic signaling pathways. The pro-apoptotic effects of Ca^2+^ are mediated by a diverse range of Ca^2+^ sensitive factors that are compartmentalized in various intracellular organelles including the ER, cytoplasm and mitochondria. The regulation of Ca^2+^ and ROS is a dynamic cyclic phenomenon as Ca^2+^ stimulates production of ROS which in turn enhances accumulation of Ca^2+^ and thus sustains this vicious cycle. Thus induction of redox imbalance by MAL-A translated into a significant increase in cytosolic Ca^2+^ (Figure 3A). When large quantities of Ca^2+^ are accumulated in the mitochondrial matrix, Ca^2+^ interacts with cyclophilin D to induce opening of the mitochondrial permeability transition pore (PTP) in the inner mitochondrial membrane which can lead to matrix swelling, rupture of the outer mitochondrial membrane and release of cytochrome *c*. Furthermore, the rise in mitochondrial Ca^2+^ stimulates the generation of factors including ROS and free fatty acids which also promotes opening of the PTP, causes dissipation of the mitochondrial membrane potential (ΔΨm) and release of Ca^2+^ [27]. MAL-A induced generation of ROS in U937 cells was accompanied by disruption of the mitochondrial membrane potential (Figure 3B). Release of cytochrome C in the cytosol in turn forms complexes with Caspase 9 and Apaf-1 (the apoptosome complex) that helps to activate executioner Caspase 3 leading to DNA fragmentation and cell death.

The negatively charged lipid cardiolipin, which has high binding affinity towards Ca^2+^ and is normally confined to the mitochondrial inner membrane, undergoes alterations during apoptosis [40]. In healthy cells, mitochondrial cytochrome *c* usually remains bound with cardiolipin and following peroxidation of cardiolipin by an oxidative assault is externalized into the cytosol [27], [40]. As MAL-A effectively peroxidised cardiolipin by 51.20%, 53.39%, and 63.29% at 1, 6 and 12 h respectively (Figure 3C), it also translated into release of free cytochrome *c* to the cytosol (Figure 3D).

Apoptosis is a natural suicidal phenomenon to control cellular growth and its abrogation leads to unregulated cellular proliferation, as observed in cancers. Apoptosis is generally classically defined by unique morphological alterations that include membrane blebbing, cytoplasmic and nuclear condensation accompanied with DNA breakage ([41] and ref. therein). An apoptotic stimulus causes externalization of phosphatidyl serine, detectable by increased binding of annexin V, a Ca^+2^ dependent phospholipid binding protein, owing to its strong affinity towards phosphatidyl serine. MAL-A effected externalization of phosphatidyl serine (Figure 3E), corroborating that MAL-A exerts its cytotoxic activity primarily via apoptosis.

Anti-cancer drug-induced apoptosis are conducted via two pathways namely an extrinsic (death receptor) or intrinsic (mitochondrial) pathway and in some cases, both pathways are involved [27]. The free cytochrome *c* in the cytosol then forms an apoptosome composed of Apaf-1 and procaspase-9, resulting in activation of caspase-9, which then activates the effector procaspases, including procaspase-3, to carry out cleavage of the DNA repair enzyme, PARP culminating in DNA degradation [42]. Executioner caspases are therefore considered critical in the apoptotic cascade and are inducible by different stimuli that include growth-factor deprivation and various environmental stresses, including anti-cancer drugs [42]. As MAL-A increased the activity of caspases-9, -3, and -8 (Figure 4A) along with PARP degradation (Figure 4C), it confirmed that MAL-A mediated its cytotoxicity by apoptosis. The role of caspases in MAL-A induced cell death was further confirmed by pre-treatment with a pan-caspase inhibitor (Z-VAD-FMK), whose ability to attenuate the cytotoxicity of MAL-A, confirmed that MAL-A mediated cytotoxicity was caspase dependent (Figure 4B). Further studies can be undertaken using either a caspase-specific blocker or preferably siRNA to delineate the specific caspases involved.

Apoptotic cells generally have active endonucleases that preferentially induce single or double stranded breaks in DNA along with chromatin condensation that translate into an increased cell population located on a DNA frequency histogram proximal to the G_0_/G_1_ peak, i.e. a sub G_0_/G_1_ peak [43]. MAL-A caused chromatin condensation (Figure 5A), increased TdT catalysed incorporation of dUTP (Figure 5B), along with apoptotic fragmentation of DNA evidenced by DNA laddering (Figure 5C), which was finally corroborated by an increased sub G_0_/G_1_ population on a DNA frequency histogram (Figure 5D, Table 2).

Taken together, MAL-A, a plant derived pro-oxidant, effectively raised the cell's oxidative status beyond a threshold limit, triggering the cell-death machinery in leukemic cells and thereby executing features of apoptosis ascribed to mammalian cells. It is anticipated that the study of the major pathways involved in MAL-A induced apoptotic death in U937 cells would provide a better insight for design of newer chemotherapeutic approaches critically needed for cancer treatment.

## References

[1] Laurent A, Nicco C, Chéreau C, Goulvestre C, Alexandre J (2005). Controlling tumor growth by modulating endogenous production of reactive oxygen species.. Cancer Res.

[2] Trachootham D, Alexandre J, Huang P (2009). Targeting cancer cells by ROS-mediated mechanisms: a radical therapeutic approach?. Nat Rev Drug Discov.

[3] Doering M, Ba LA, Lilienthal N, Nicco C, Scherer C (2010). Synthesis and selective anticancer activity of organochalcogen based redox catalysts.. J Med Chem.

[4] Beck R, Dejeans N, Glorieux C, Pedrosa RC, Vásquez D (2011). Molecular chaperone Hsp90 as a target for oxidant-based anticancer therapies.. Curr Med Chem.

[5] Zhou Y, Hileman EO, Plunkett W, Keating MJ, Huang P (2003). Free radical stress in chronic lymphocytic leukemia cells and its role in cellular sensitivity to ROS-generating anticancer agents.. Blood.

[6] Yang ES, Choi MJ, Kim JH, Choi KS, Kwon TK (2011). Combination of withaferin A and X-ray irradiation enhances apoptosis in U937 cells.. Toxicol In Vitro.

[7] Morita T, Jinno K, Kawagishi H, Arimoto Y, Suganuma H (2003). Hepatoprotective effect of myristicin from nutmeg (*Myristica fragrans*) on lipopolysaccharide/d-galactosamine-induced liver injury.. J Agric Food Chem.

[8] Banerjee D, Bauri AK, Guha RK, Bandyopadhyay SK, Chattopadhyay S (2008). Healing properties of malabaricone B and malabaricone C, against indomethacin-induced gastric ulceration and mechanism of action.. Eur J Pharmacol.

[9] Sen R, Bandyopadhyay S, Dutta A, Mandal G, Ganguly S (2007). Artemisinin triggers induction of cell-cycle arrest and apoptosis in *Leishmania donovani* promastigotes.. J Med Microbiol.

[10] Patro BS, Bauri AK, Mishra S, Chattopadhyay S (2005). Antioxidant activity of *Myristica malabarica* extracts and their constituents.. J Agric Food Chem.

[11] Patro BS, Tyagi M, Saha J, Chattopadhyay S (2010). Comparative nuclease and anti-cancer properties of the naturally occurring malabaricones.. Bioorg Med Chem.

[12] Jaeger T, Flohe L (2006). The thiol-based redox networks of pathogens: unexploited targets in the search for new drugs.. Biofactors.

[13] Saha P, Mukhopadhyay D, Chatterjee M (2011). Immunomodulation by chemotherapeutic agents against Leishmaniasis.. Int Immunopharmacol.

[14] Sen R, Chatterjee M (2011). Plant derived therapeutics for the treatment of Leishmaniasis. Phytomedicine..

[15] Paris C, Bertoglio J, Bréard J (2007). Lysosomal and mitochondrial pathways in miltefosine-induced apoptosis in U937 cells.. Apoptosis.

[16] Verma NK, Dey CS (2004). Possible mechanism of miltefosine-mediated death of *Leishmania donovani*.. Antimicrob Agents Chemother.

[17] Guha P, Dey A, Sen R, Chatterjee M, Chattopadhyay S (2011). Intracellular GSH depletion triggered mitochondrial Bax translocation to accomplish resveratrol-induced apoptosis in the U937 cell line.. J Pharmacol Exp Ther.

[18] Bhattacharya K, Samanta SK, Tripathi R, Mallick A, Chandra S (2010). Apoptotic effects of mahanine on human leukemic cells are mediated through crosstalk between Apo-1/Fas signaling and the Bid protein and via mitochondrial pathways.. Biochem Pharmacol.

[19] Ganguly S, Bandyopadhyay S, Sarkar A, Chatterjee M (2006). Development of a semi-automated colorimetric assay for screening anti-leishmanial agents.. J Microbiol Methods.

[20] Sarkar A, Saha P, Mandal G, Mukhopadhyay D, Roy S (2011). Monitoring of intracellular nitric oxide in leishmaniasis: its applicability in patients with visceral leishmaniasis.. Cytometry A.

[21] Sarkar A, Mandal G, Singh N, Sundar S, Chatterjee M (2009). Flow cytometric determination of intracellular non-protein thiols in *Leishmania* promastigotes using 5-chloromethyl fluorescein diacetate.. Exp Parasitol.

[22] Rotruck JT, Pope AL, Ganther HE, Swanson AB, Hafeman DG (1973). Selenium: biochemical role as a component of glutathione peroxidase.. Science.

[23] Saha P, Sen R, Hariharan C, Kumar D, Das P (2009). Berberine chloride causes a caspase-independent, apoptotic-like death in *Leishmania donovani* promastigotes.. Free Radic Res.

[24] Saha P, Bhattacharjee S, Sarkar A, Manna A, Majumder S (2011). Berberine chloride mediates its anti-leishmanial activity via differential regulation of the mitogen activated protein kinase pathway in macrophages.. PLoS One.

[25] Munoz MA, Pacheco A, Becker MI, Silva E, Ebensperger R (2011). Different cell death mechanisms are induced by a hydrophobic flavin in human tumor cells after visible light irradiation.. J Photochem Photobiol B.

[26] Kim PK, Zamora R, Petrosko P, Billiar TR (2001). The regulatory role of nitric oxide in apoptosis.. Int Immunopharmacol.

[27] Orrenius S, Zhivotovsky B, Nicotera P (2003). Regulation of cell death: the calcium-apoptosis link.. Nat Rev Mol Cell Biol.

[28] Barbier M, Gray BD, Muirhead KA, Ronot X, Boutonnat J (2004). A flow cytometric assay for simultaneous assessment of drug efflux, proliferation, and apoptosis.. Cytometry B Clin Cytom.

[29] Nomura K, Imai H, Koumura T, Arai M, Nakagawa Y (1999). Mitochondrial phospholipid hydroperoxide glutathione peroxidase suppresses apoptosis mediated by a mitochondrial death pathway.. J Biol Chem.

[30] Das B, Chowdhury C, Kumar D, Sen R, Roy R (2010). Synthesis, cytotoxicity, and structure–activity relationship (SAR) studies of andrographolide analogues as anti-cancer agent.. Bioorg Med Chem Lett.

[31] Newman DJ, Cragg GM, Snader KM (2000). The influence of natural products upon drug discovery.. Nat Prod Rep.

[32] Pelicano H, Carney D, Huang P (2004). ROS stress in cancer cells and therapeutic implications Drug Resist Updat.

[33] Circu ML, Aw TY (2010). Reactive oxygen species, cellular redox systems, and apoptosis.. Free Radic Biol Med.

[34] Storz P (2005). Reactive oxygen species in tumor progression.. Front Biosci.

[35] Cadenas E (2004). Mitochondrial free radical production and cell signaling.. Mol Aspects Med.

[36] Brockhaus F, Brune B (1998). U937 apoptotic cell death by nitric oxide: Bcl-2 downregulation and caspase activation.. Exp Cell Res.

[37] Schnelldorfer T, Gansauge S, Gansauge F, Schlosser S, Beger HG (2000). Glutathione depletion causes cell growth inhibition and enhanced apoptosis in pancreatic cancer cells.. Cancer.

[38] Aruoma OI, Halliwell B, Hoey BM, Butler J (1989). The antioxidant action of N- acetylcysteine: its reaction with hydrogen peroxide, hydroxyl radical, super- oxide, and hypochlorous acid.. Free Radic Biol Med.

[39] Singh SV, Srivastava SK, Choi S, Lew KL, Antosiewicz J (2005). Sulforaphane-induced cell death in human prostate cancer cells is initiated by reactive oxygen species J Biol Chem.

[40] Tyurina YY, Kini V, Tyurin VA, Vlasova II, Jiang J (2006). Mechanisms of cardiolipin oxidation by cytochrome *c*: relevance to pro and antiapoptotic functions of etoposide.. Mol Pharmacol.

[41] Harada H, Grant S (2003). Apoptosis regulators.. Rev Clin Exp Hematol.

[42] Zou H, Yang R, Hao J, Wang J, Sun C (2003). Regulation of the Apaf-1/caspase-9 apoptosome by caspase-3 and XIAP.. J Biol Chem.

[43] Sanchez CA, Rodriguez E, Varela E, Zapata E, Paez A (2008). Statin-induced inhibition of MCF-7 breast cancer cell proliferation is related to cell cycle arrest and apoptotic and necrotic cell death mediated by an enhanced oxidative stress.. Cancer Invest.

